# The Creation of Surrogate Models for Fast Estimation of Complex Model Outcomes

**DOI:** 10.1371/journal.pone.0156574

**Published:** 2016-06-03

**Authors:** W. Andrew Pruett, Robert L. Hester

**Affiliations:** Department of Physiology and Biophysics, Center for Computational Medicine, University of Mississippi Medical Center, Jackson, Mississippi, United States of America, 39215; Fraunhofer Research Institution of Marine Biotechnology, GERMANY

## Abstract

A surrogate model is a black box model that reproduces the output of another more complex model at a single time point. This is to be distinguished from the method of surrogate data, used in time series. The purpose of a surrogate is to reduce the time necessary for a computation at the cost of rigor and generality. We describe a method of constructing surrogates in the form of support vector machine (SVM) regressions for the purpose of exploring the parameter space of physiological models. Our focus is on the methodology of surrogate creation and accuracy assessment in comparison to the original model. This is done in the context of a simulation of hemorrhage in one model, “Small”, and renal denervation in another, HumMod. In both cases, the surrogate predicts the drop in mean arterial pressure following the intervention. We asked three questions concerning surrogate models: (1) how many training examples are necessary to obtain an accurate surrogate, (2) is surrogate accuracy homogeneous, and (3) how much can computation time be reduced when using a surrogate. We found the minimum training set size that would guarantee maximal accuracy was widely variable, but could be algorithmically generated. The average error for the pressure response to the protocols was -0.05±2.47 in Small, and -0.3 +/- 3.94 mmHg in HumMod. In the Small model, error grew with actual pressure drop, and in HumMod, larger pressure drops were overestimated by the surrogates. Surrogate use resulted in a 6 order of magnitude decrease in computation time. These results suggest surrogate modeling is a valuable tool for generating predictions of an integrative model’s behavior on densely sampled subsets of its parameter space.

## Introduction

The pharmaceutical sector is the largest component of the biosciences industry, but it faces a critical challenge. Research and development costs increased from $15 to $85 billion between 1995 and 2010. The number of new compounds approved by FDA has fallen from around 50 per year in the 1990s to currently 20 per year. Attrition rates have increased from 20% to 50% between 1990 and 2004, with the majority of failures being due to lack of efficacy. Less than 10% of compounds entering a clinical trial are ultimately introduced to the market [1]. This suggests that the current model of pharmaceutical and device development and testing is subject to continued growing expense and decreased efficiency.

A potential solution to these challenges lies in modeling and simulation. Examples of the use of simulation in clinical trials are reviewed in Chapter 10 of [2]. However there are significant problems in predicting the human response to medical therapy. Humans have a wide range of responses to the same stimulus, brought on by anatomic, biochemical, or genetic differences between them. Physiology is composed of multiple feedback loops organized in a complex network of control systems. The fundamental physiological and biochemical relationships are the same in all individuals, but small differences in the magnitude or sensitivity of these control systems to a perturbation will propagate throughout the system, yielding enormous inter-individual differences.

Physiology based modeling (PBM), which links biochemical and physical relationships in a mechanistic way, allows this concept to be developed more fully. In PBM, models are developed around the hypothetical mechanisms of tissue responses and system controls. The collection of equations of these mechanisms, with coefficients treated as additional variables (parameters), is henceforth referred to as a *generic model*. Parameters can then be identified with physiological quantities, and responses correlated with parameters. Instantiating a generic model with a parameter set (*virtual patient*) allows treatment options to be explored in the confines of the model, and clinical and experimental hypotheses to be tested. In this setting, individuals are represented as different parameter sets, and the differences between individuals are encoded by their parameters. The original version of this type of modeling was the Guyton/Coleman/Granger model, currently branded as HumMod [3, 4]. This model is currently comprised of ~8000 variables and parameters describing integrative physiology. Other groups have pursued similar philosophies of modeling, such as the Virtual Physiological Human [5], The Physiome Project [6], or the Virtual Physiological Rat project [7].

We have previously shown that replacing a deterministic mechanistic model with a cohort of similar models can generate a plausible population response after hemorrhage [8]. There, an algorithm was used to simultaneously calibrate multiple model independent variables to create a population of individual models whose response matched that of a previously published experiment. Formally, this type of problem is referred to as an inverse problem. The method that we used was too slow to be useful in practice. Given *n* degrees of freedom, with *m* samples from each dimension, Latin square sampling requires *m*^*n-1*^ samples. This is intractable for even small values of *m* and *n*. Our study demonstrated that the inverse problem could be broken into two pieces of great complexity. The first challenge is to create enough virtual patients and their outputs in a reasonable amount of time, and the second challenge is to calibrate the model to match a given population in one or more endpoints. In the current paper we propose a solution for the sampling challenge by utilizing surrogate models, which rapidly estimate a single model output using a machine-learning generated black box model.

A surrogate model reduces the complexity of the integrative model with respect to a specific endpoint. The surrogate model does not need to be informed of the internal characteristics of the model: it is a black box that simply mirrors the model outputs in a limited context. For example, one could derive from a computationally complex model of a skeletal muscle contracting a much simpler model of the muscle’s contraction that yielded useful output linking work levels to local tissue blood flow and metabolic activity. That same surrogate would not necessarily be a good model for a larger version of the same muscle, due to differences in internal flows and stresses in a new geometry, or in a muscle after training due to changes in how energy is produced, stored, and utilized in trained muscles. However it might be a good estimator of blood flow to a normal muscle at a variety of pressure and workloads. This illustrates the essential feature of a surrogate: a tradeoff between a wide context of use and large computational time for a narrow context but faster computation time. Surrogate model methods are common in areas that require a large number of computations, including airfoil dynamics [9], supply chain management [10], and material science [11].

In this paper, we describe a method of constructing surrogate models for the purpose of exploring two physiology-based mathematical models: a small circulatory model (Small) and HumMod. Our focus in this paper is on the methodology of surrogate creation and accuracy assessment in comparison to the original model. We considered distinct protocols in the two models to illustrate the utility of this methodology in creating efficient surrogates. In both cases, we created a surrogate that mapped a parameter sample to an expected fall in arterial pressure at a specific time point after an intervention. First, we estimated the response of the Small model with respect to a fixed hemorrhage. While hemorrhage is not a therapy, it is a perturbation that induces a system-wide physiological response, and has a correspondingly wide range of responses in normal humans [12, 13]. Second, we consider the effect of partial renal denervation on mean arterial pressure in HumMod. Recent Symplicity clinical trials demonstrate a large variability in response to this therapy but the mechanisms are unknown [14, 15]. Preliminary HumMod simulations of these clinical trials by varying the parameters in a large virtual population yielded the same variability as the patients in the trial, suggesting that the responses could be predicted. In this study, we provide a method for generating a heterogeneous population for use in calibrating or validating a model.

## Materials and Methods

### Description of Models

**Fig 1:**
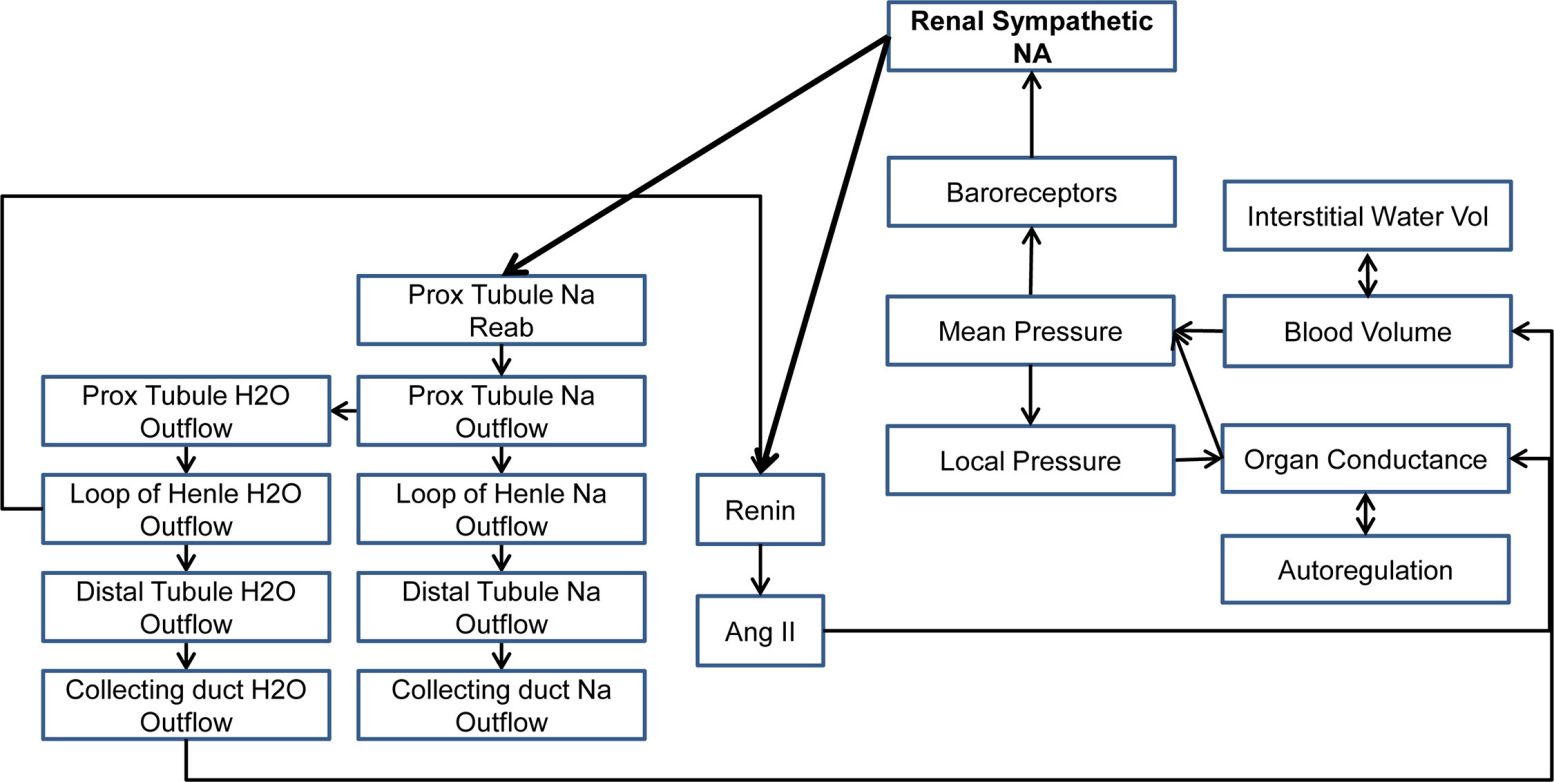
HumMod and renal denervation. A summary of the systems implicated in the body’s response to renal denervation, as modeled in HumMod.

For this study, we utilized two integrative physiology based mathematical models. The first, Small, a small circulatory model used for education purposes, will be exposed to a fixed hemorrhage. Small is a minimal model of 26 parameters and 26 variables, linking sympathetic nerve activity, the Starling cardiac output curve, the venous return curve, a simple kidney, and stressed/unstressed fluid volumes, used to simulate hypervolemia/hypovolemia and congestive heart failure. The model is highly nonlinear, and relies on a solution of the equation “cardiac output = venous return” at each time step, preventing more typical analysis with linear algebra or differential geometry. The model has a basal parameterization replicating an average adult male. The second model, HumMod, is a mixture of algebraic, differential, and implicit equation methods spanning 14 organ systems, linked with circulatory, neural, and endocrine systems. An overview of the systems involved in HumMod are shown in Fig 1. The mixed modeling techniques as well as the overall nonlinearity of HumMod present a unique challenge from the analysis perspective. HumMod is a deterministic model that has been calibrated to behave like a 37 year old man in good health. For both models, we refer to the native calibration as the “typical” model throughout, and for each parameter, its value in the typical model is referred to as its typical value.

### Overview of Method

The process we describe below required several steps. First, a *sensitivity analysis* was used to reduce the total number of model parameters that were allowed to vary down to the ones that were sensitive (i.e. the parameters that had the most influential effects in the model). Next, parameter vectors were sampled from a uniform distribution about the baseline value of each of the sensitive parameters. Instantiating the model with these parameter samples yielded virtual patients we termed *primer models*. These virtual patients were brought to steady state, subjected to an intervention, and their response was observed. These responses, along with the parameter vectors that created the patients, were partitioned into “training” and “testing” populations. Each training population was used to create a surrogate model that would predict the response of a new virtual patient (parameter vector) to the same intervention. This prediction was then evaluated and validated on the testing population.

### Sensitivity Analysis

Changing the model parameters generates a new steady state for the model, which we interpret as a new virtual patient. Only a limited collection of parameters are influential to the response for a given intervention. To determine what these influential parameters might be, we utilized a sensitivity analysis, testing specifically for first order effects. Because of the presence of interactions between parameters, all parameters (400 in the case of HumMod, 26 in the case of Small) were allowed to vary simultaneously within 10% of their original values. Two hundred samples were drawn; in each case, simulations were run until the model was settled, and the variables of interest were observed in each virtual patient. For the Small model, these endpoints were extracellular fluid volume (ECFV), blood volume (BV), mean arterial pressure (MAP), and cardiac output (CO). For HumMod, the only output of interest was mean arterial pressure. The outputs of interest were chosen, rather than determined algorithmically. Each parameter *p* exhibited a mean *μ*_*p*_ and standard deviation *σ*_*p*_ in the virtual patient population. For a threshold *δ*, we partitioned the population into Θ_<_, the patients whose sampled value for *p* lay within *δσ*_*p*_ of *μ*_*p*_ (“near” models), and Θ_>_, the remainder of patients (“far” models). Denoting an output of interest, *y*, by $y(\theta_{\hat{p}})$ to indicate that *y* is the value of the virtual patient whose sample parameter vector is *θ*, with $\hat{p}$ the sampled value for *p*, we calculated:

$$r_{p,\delta }≔\frac{Var(y(\theta_{\hat{p}}):\;\theta_{\hat{p}}\;in\;\Theta_{>})}{Var(y(\theta_{\hat{p}}):\;\theta_{\hat{p}}\;in\;\Theta_{<})}.$$

Hence, when “far” values of the parameter induce greater variance than “near” values relative to *δ*, the ratio increases, indicating great influence of *p* on the model outcome. This ratio was computed for (*δ* = 0.1, 0.2, 0.3, 0.4, 0.5, 0.75, 1, 1.25), and a ratio value over 1.1 was used as a threshold for sensitivity. Those parameters that were influential at more than four values of *δ* were considered persistent (or sensitive) and were allowed to vary when creating the primer models. All other parameters were held constant at their baseline value in the primer models.

From these, we created surrogate models. This task had three steps: sampling a primer set to train the machine learning algorithm, creation of the support vector machines (SVM) to generate surrogate models, and validating the surrogate models against outputs from the actual model (either Small or HumMod).

### Primer Models for Supervision of Surrogates

For both Small and HumMod, primer models (virtual patients) were created by varying the influential parameters (for each of the 2000 individuals). Each parameter from each individual was created by randomly drawing from a uniform distribution centered on the baseline parameter values, with a radius of 10% of the parameter’s baseline value. These individuals were brought to steady state by simulating 1 week, and then were subjected to their intervention. For the Small model, the intervention was hemorrhage of 50 mL/min for 20 minutes. Preliminary simulations indicated that this rate and amount would yield, on average, a 20 mmHg drop in pressure. For HumMod, each individual was given a purely sympathetic hypertension resulting from an increased sympathetic tone to the kidneys and heart to imitate sleep apnea-associated hypertension. After steadying, the HumMod primer models were subjected to 40% decrease in sympathetic outflow to the kidneys, a change consistent with the renal denervation in clinical trials [16]. Preliminary simulations suggested the majority of the blood pressure effect of renal denervation was within two weeks. Hence the models were allowed two weeks to reach steady state, and the difference in mean pressure was measured. The parameter vector from each virtual individual, along with the variable rosters, model outputs, and Mathematica code used for analysis, are available at https://github.com/drew-pruett/Surrogates. Additionally, the Small model and an early version of HumMod are freely available at https://github.com/HumMod/small-stoch-model and https://github.com/HumMod/hummod-standalone, respectively. Up to date versions of HumMod can be requested for free academic use.

### Surrogate Construction and Analysis

SVM surrogates were constructed using subsets of the 2000 individuals. The SVM are regression models mapping vectors of parameter samples to pressure drops after insult. The SVM were constructed using svm-light (www.svm-light.joachims.org) [17]. All kernels were radial-basis kernels of the form

$$K(x,y)=e^{-\gamma \cdot (\left|\left|x\right||-|\left|y\right|\right|)}.$$

This designation followed intuitively from the similarity of the radial basis function to a low pass smoothing filter. We allowed only sensitivity (γ) and the slack coefficient as parameters of the SVM fitting process. The slack coefficient controls the tradeoff between the size of the region where mistakes are allowed and the number of mistakes in that region.

The parameter vectors used to generate the virtual individuals were normalized (Z-scores) and had the unaltered pressure drop appended. This collection of vectors was randomly sampled to create collections θ_n_ of individuals for training populations of different size, where *n* = 25, 50, 75, 100, 125, 150, 250, 500, and 750 for the Small model, and *n* = 200, 400, 600, 800, 1000, 1250, and 1500 for HumMod. These were used to generate a series of SVM indexed by *n*. For the n^th^ training population θ_n_, its complement was used to test the accuracy of the SVM associated with *n*.

For both the Small Model and the HumMod experiments, the support vector machine functioned as a regression equation, and accuracy was assessed by comparing SVM prediction with the Small Model or HumMod outputs. Raw error (difference in predicted pressure fall between the integrative model and surrogate) was computed for all virtual patients in each experiment. A bias in surrogate behavior at some locations could conceivably be obfuscated by opposite bias in other locations. To address this issue, we used rolling average errors. For a given pressure drop Δ*P* and radius *r*, we computed the error of all virtual patients whose model predicted they would fall in the window (Δ*P* − *r*,Δ*P* + *r*).

## Results

We began with a sensitivity analysis in each model as described above. The results are shown in Table 1 and Table 2.

**Table 1:** Parameters in Small sensitive to hemorrhage.

Sensitivity of afferent sympathetics to baroreceptor	Fluid intake	Sensitivity of compliance to sympathetic nerve stimulation
Minimal efferent sympathetic nerve activity	Sensitivity of urine output to changes in pressure	Maximum blood part of blood/ECFV partition
Unstressed fluid volume	Set point of autoregulation	Set point of blood volume in blood/ECFV partition

**Table 2:** Parameters in HumMod sensitive to renal denervation.

Left Atrial Pressure set point	ANP Secretion Right Atrium base	Unstressed fluid volume-large vein	Right heart contractility base
Right Atrial Pressure set point	ANP Secretion Left Atrium base	Sensitivity of Sympathetic effect on PT Na reabsorption	Left heart contractility base
Collecting Duct Na basic fraction	Ang-II receptor set point-Proximal tubule	Set point of Sympathetic effect on PT Na reabsorption	Base renin synthesis
Distal tubule Na basic fraction	Ang-II receptor minimal stimulation level-proximal tubule	Sensitivity of renin secretion to sympathetics	Sensitivity of renin synthesis to sympathetics
Loop Na basic fraction	Ang-II receptor sensitivity level-proximal tubule	Minimal effect of secretion on renin synthesis	Minimal effect of sympathetics on renin synthesis
Proximal tubule Na basic fraction			

**Fig 2:**
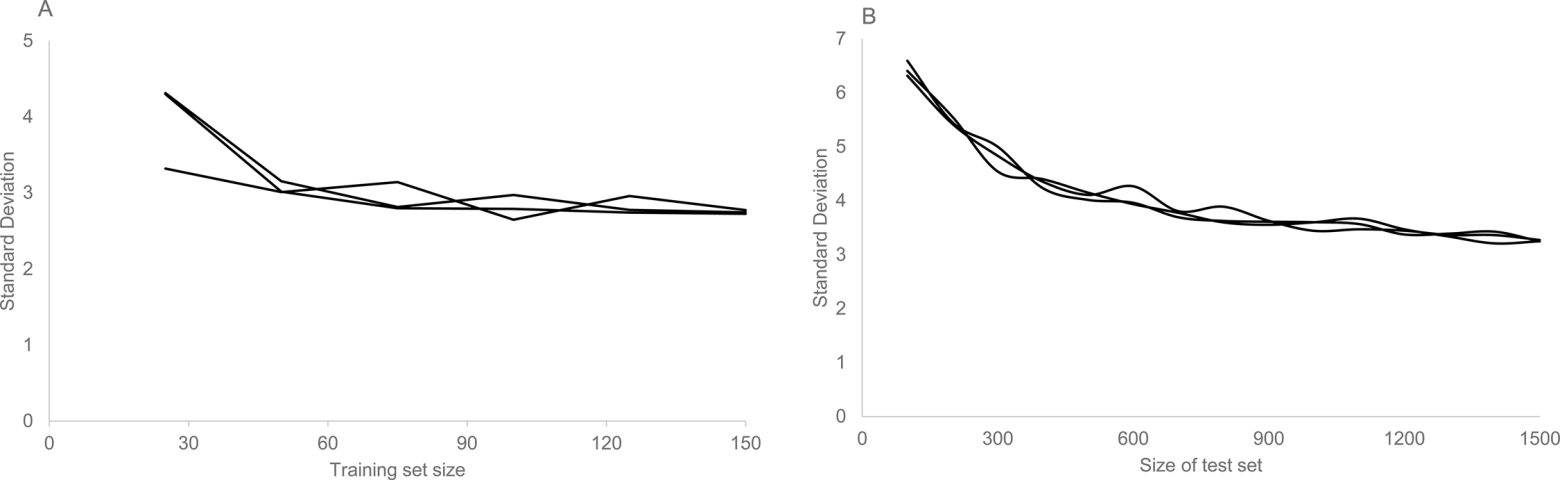
Error in pressure change as a function of training set size. A primary goal of this work was to understand the size of the training set necessary to give an accurate prediction of integrative model behavior. We examined Small, using 25 to 150 virtual patients in steps of 25, comparing model and surrogate pressure drops in three trials. The standard deviation of the error was plotted against the training set size (A). Using the same process, we compared HumMod and its surrogate using 100 to 1500 virtual patient in steps of 100 (B). In both cases, the standard deviation settled to a stable quantity.

The primary goal of this paper is to compare the performance of surrogate models constructed from different sizes of training sets to the original physiological model for the purpose of rapidly sampling model parameter space. To find the minimal training set size necessary to predict integrative model outputs with maximum accuracy, we increased training set size until the standard deviation of the error stabilized. As the associated error with the surrogate process ceased to change, the point of stabilization was assumed to denote the minimum number of models necessary for a maximally accurate surrogate. For Small we used steps of 25; the standard deviation of the error stabilized at 3 mmHg at *n* = 100. For HumMod, we used steps of 100 for the increase, and standard deviation of the error stabilized to 3.6 mmHg at *n* = 900. These details are shown in Fig 2.

**Fig 3:**
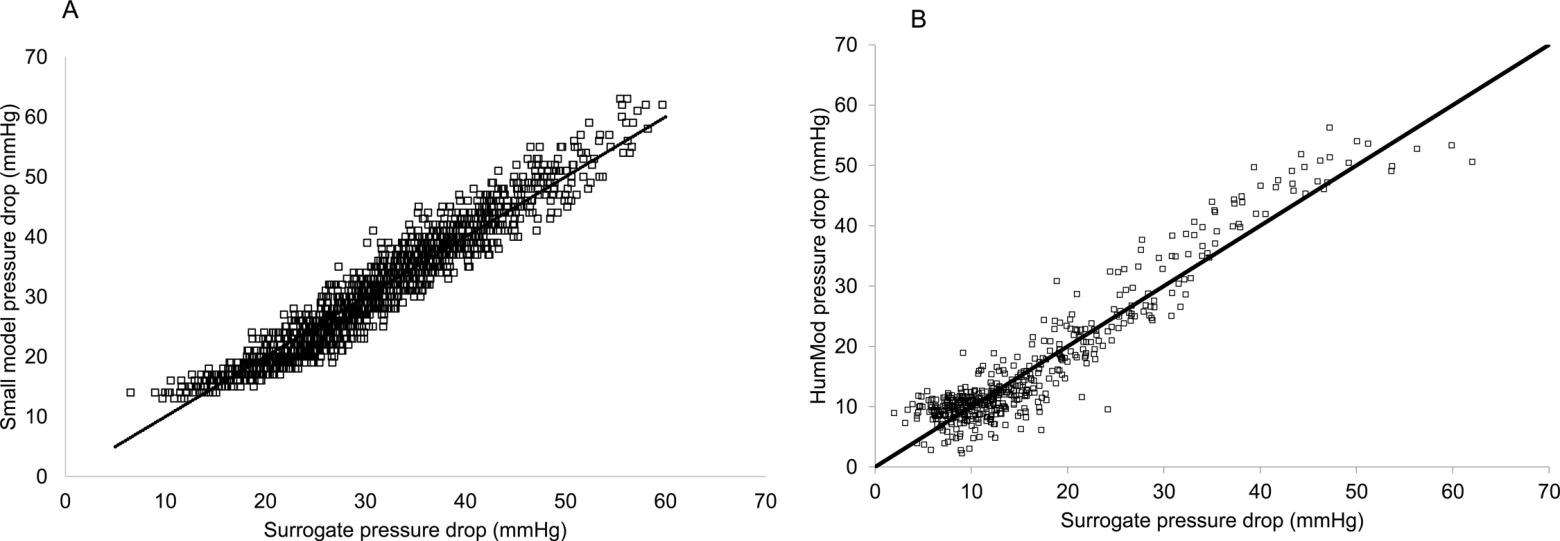
Pressure differences in the testing set in HumMod and the regression surrogate. A scatterplot showing the differences in baseline and post-intervention pressures in integrative model (vertical axis) and surrogate predictions built from 1000 members of the primer population. Small is shown in A, and HumMod in B. The differences are normally distributed with mean -0.1 and standard deviation 2.95 for the small model, and mean -0.3 with standard deviation 3.94 for HumMod. The predictions and model outputs were distributed similarly (p<0.05).

The comparison of surrogate regressions and integrative models at a single population size (n = 1000) are shown in Fig 3, along with the histogram of errors for both models. There are statistically no differences between the surrogates and the integrative models in either case, with the difference in a decrease in pressure equal -0.05±2.47 in the Small hemorrhage protocol and -0.3±3.94 mmHg for the HumMod/renal denervation protocol. This indicated that the surrogates are a good estimators of these two integrative models.

**Fig 4:**
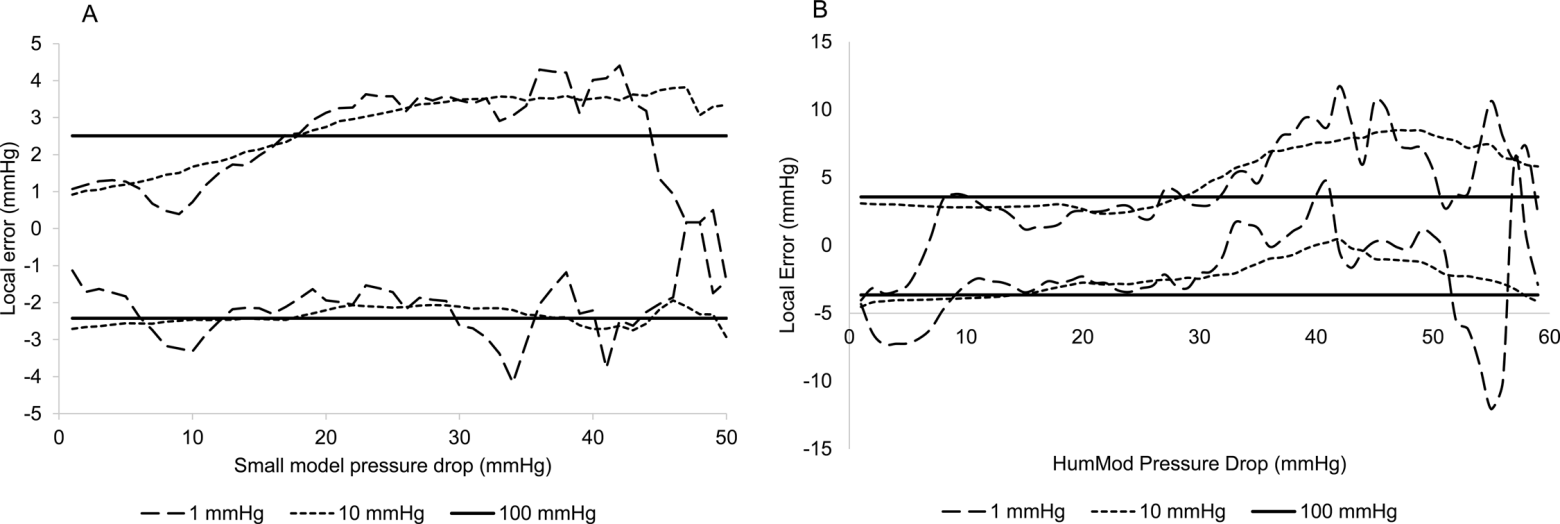
Rolling average error. The rolling average error between integrative model and surrogates is computed for 1, 10, and 100 mmHg radii (dashed, dotted, and solid lines, respectively) in Small (3A) and HumMod cases (3B). In both cases, there is a tendency to bias towards negative error at small pressure drops, and towards positive error in large pressure drops.

The next significant question was to address where surrogate errors occurred. This was assessed with the rolling error method. Three radii were chosen: 1, 10, and 100 mmHg. The smallest radius gave a very fine grained view of error, the 10 mmHg radius gave a much wider smoother error approximation, and the 100 mmHg radius assessed global error. The results are shown in Fig 4A (Small) and 4B (HumMod). Small shows very similar behavior in the 1 and 10 mmHg windows, with a bias towards negative error at low predicted pressure drops, and a bias towards positive errors at large predicted pressure changes. The bias is around 2 mmHg. In the surrogate for HumMod, the 1 mmHg window shows more fluctuation, especially at very large predicted pressure changes. As before, this bias is towards negative error at small predicted pressure changes, and towards positive error in large changes, but the bias is insignificant. The 10 mmHg window shows systematic 5 mmHg positive bias in high ranges.

Another goal of this paper is to create a technique that is able to more rapidly determine the relationship between pre-defined endpoints and model parameters. For the primer population of 2000 individuals, the two integrative models were run via scripts on two identical Dell Windows 7 desktop computers. On average, Small took 26 seconds for each pass through the settling and hemorrhage protocol, while HumMod took ~5 minutes to complete the 4 week script of settling, intervention, and resettling. Construction of the surrogates took less than 10 seconds in each case. Once the surrogates were created, sampling of 1000000 parameter sets and transforming them with the surrogate took approximately 1 minute in either Small or HumMod case, a reduction in sample-to-output time of 6 orders of magnitude in both cases. The majority of the sampling time is spent writing the file for svm-light to execute.

## Discussion

Variability in human responses complicates the interpretation of data in clinical and experimental situations. This is a common problem in complex dynamical systems, and has been addressed in other contexts. In engineering especially, complex systems must be analyzed for failure modes: points at which the system can fail catastrophically. As an example, an engineer builds models of wings that vary from the basic design and tests all of them in a simulated wind tunnel. In this paper, we describe the same type of endeavor.

Physiologically-based modeling begins with mechanisms that are well studied in controlled settings in the clinical setting or laboratory. Because the mechanisms we model can be linked to established physics or chemistry, equations that model those mechanisms can be reliably described. Linking well-understood mechanisms together yields chains of models that interact and integrate to form a more complex model with emergent properties that arise from those interactions. The question of how best to describe those emergent properties is one that we address here. Classically, a methodology such as systems identification might be used to construct a statistical link between inputs and outputs. This technique is very robust to situations in which incomplete information exists concerning the system being studied. The current efforts reflect a different approach, using machine learning and feature space regression instead. We believe that this methodology is entirely appropriate because of the mechanistic basis of the models we consider.

In order to simulate the response of a therapy on humans, a mathematical model must be able to simulate many intersecting control systems in order to predict efficacy and safety, rather than just the target of the agent. Given a mathematical model of those control systems and the dose response of the therapy on those control systems, a model for the therapy may be hypothesized. Accurate prediction of the mean response to intervention may be useful, but it falls far short of the goal of personalized therapy. It is not enough to simulate the relevant control systems and feedback loops for an ideal individual; an entire population must be simulated. We suggest that a useful mathematical model must rely on modeling mechanisms, rather than empirical observations. Variation in mechanistic parameters naturally reflects inter-individual differences. The process can be a time-consuming endeavor and prevent effective sampling of the parameter space. Some scalable methodology is necessary for this task. We propose a surrogate modeling technique in parallel with those used in computationally intensive problems in other disciplines.

An important component of this study was to determine the size of the training set required to generate a maximally predictive surrogate. The number of parameters was similar in the two models, but Small required far fewer training examples (100) for the supervised learning process than did HumMod (900). HumMod is an enormously complex model with many more control systems that appear in Small. These data suggests that these interactions likely drive up surrogate complexity, requiring more samples for accurate estimation. To avoid this excess in the future, we suggest constructing collections of individuals of increasing size, training on half and testing on the remainder. When the variance in error approaches a constant value, as in Fig 1, the process can terminate.

A critical question in this study was to identify the best way to assess the surrogate performance against the models used. The condition that errors be small was necessary, but not sufficient. Having significant errors in some regions and no error in others can lead to a low average error, but this does not ensure good performance. Instead, we used a rolling average assessment of error at three different radii. This allowed us to search for both very local and more general biases in the surrogate representations of their respective models. In both Small and HumMod, surrogates were accurate but tended to under-predict pressure drops at smaller pressures, and over predict the size of a pressure drop when the integrative model pressure drop was large. However, this bias was not significant, never reaching more than 5 mmHg. From this, we see that the surrogates accurately estimate model behavior across a wide range of responses.

Finally, this study showed conclusively that SVM surrogates were capable of efficiently estimating the response of large numbers of virtual patients in a short time. Creation of a surrogate from a collection of supervising primer models was a matter of less than one minute, regardless of training set size or integrative model. Once the surrogate model was determined, generating one million new virtual patients and their responses required less than one minute on a desktop computer. This represents an enormous ability to scale up the production of parameter-outcome pairs and is a necessary addition to the model calibration toolbox.

There are several limitations for this technique. First, the surrogate estimates are limited by the accuracy and validation of the integrative model. Second, the reduction in time required to obtain outcomes from parameter samples is limited to about 6 orders of magnitude. Hence the number of parameters that can be densely sampled simultaneously is still restricted. Finally, training and testing sets must be generated within the model. Although the size of these sets was reasonably bounded in the two cases we specified, we do not understand why such a large discrepancy was observed between the two. It is possible that a more complicated model would require larger training and testing sets than could be feasibly constructed.

For future studies, this technique makes it possible to investigate the efficacy of different calibration techniques on large physiologically based models. Such models have large numbers of parameters that can markedly influence a particular outcome. Dense sampling in many degrees of freedom with a complex and time-consumptive mathematical model is an intractable problem. Here we show that a small number of primer models can effectively train the surrogates, making dense sampling a tractable problem. Our techniques afford researchers the capability to scale up a small number of computationally expensive primers into an unlimited collection of inexpensive, accurate model predictions.

In clinical trials or in patient care, the heterogeneity in individual response to interventions begs some level of study. Monogenic or single source causes such as genetic differences have been used with great effect to explain variability in some cases [18]. However, in hemorrhage, multiple factors have been shown to weakly correlate with response [19] but the lack of sensitivity or specificity of response to these individual causes suggest a deeper root cause of variability. Our previous work indicates the interactions of multiple parameters in predicting the blood pressure drop at a specific level of hypovolemia [8], but a fuller analysis was impossible due to the speed of the model. Similarly, preliminary studies indicated that variation in the pressure drop following renal denervation could be attributed to parameter variability, but the physiology could not be adequately explored with the tools available at that time. The surrogate approach will be valuable moving forward in both of these contexts as we seek to understand the physiology underlying inter-individual variation.

## Conclusion

In this paper, we present a technique for approximating the outcome of a complex physiological modeling with a nonlinear regression obtained through machine learning methods. We demonstrate that, in our example, 900 samples were sufficient to generate highly accurate predictions of outcomes to renal denervation at a wide range of thresholds in HumMod, and that 100 samples was enough to accurately predict hemorrhage outcomes in Small. Our results suggest that this technique can be used to deeply sample high dimensional parameter spaces for the purpose of understanding how small differences in the coefficients of models can lead to robust and variable responses. Because the model we approximated is physics based, future work should include parameter variation that can be identified with real physiological and anatomical differences, giving further insight into the processes behind human variability.

To use a PBM in a clinical environment, it must be rigorously validated against the population it purports to describe, and its domain of applicability must be specified. We believe that this tool will be useful in this process for a wide variety of models, allowing models to be tested for robustness and for higher degree sensitivity analyses.

## References

[1] Topol EJ. The creative destruction of medicine: how the digital revolution will create better health care New York: Basic Books; 2012. xi, 303 p. p.

[2] Combs CD, Sokolowski JA, Banks CM. The digital patient: advancing healthcare, research, and education Hoboken, New Jersey: John Wiley & Sons Inc.; 2016. p. p.

[3] Guyton AC, Coleman TG, Cowley AV Jr., Scheel KW, Manning RD Jr., Norman RA Jr. Arterial pressure regulation. Overriding dominance of the kidneys in long-term regulation and in hypertension. Am J Med. 1972;52(5):584–94. 4337474 10.1016/0002-9343(72)90050-2

[4] Hester RL, Brown AJ, Husband L, Iliescu R, Pruett D, Summers R, et al HumMod: A Modeling Environment for the Simulation of Integrative Human Physiology. Front Physiol. 2011;2:12 10.3389/fphys.2011.00012 21647209 PMC3082131

[5] Viceconti M, Kohl P. The virtual physiological human: computer simulation for integrative biomedicine I. Philos Trans A Math Phys Eng Sci. 2010;368(1920):2591–4. 10.1098/rsta.2010.0096 20439263

[6] Hunter PJ, Borg TK. Integration from proteins to organs: the Physiome Project. Nat Rev Mol Cell Biol. 2003;4(3):237–43. 12612642 10.1038/nrm1054

[7] Beard DA, Neal ML, Tabesh-Saleki N, Thompson CT, Bassingthwaighte JB, Shimoyama M, et al Multiscale modeling and data integration in the virtual physiological rat project. Ann Biomed Eng. 2012;40(11):2365–78. 10.1007/s10439-012-0611-7 22805979 PMC3463790

[8] Pruett WA, Husband LD, Husband G, Dakhlalla M, Bellamy K, Coleman TG, et al A population model of integrative cardiovascular physiology. PLoS One. 2013;8(9):e74329 10.1371/journal.pone.0074329 24058546 PMC3772858

[9] Jeong S, Murayama M, Yamamoto K. Efficient optimization design method using kriging model. Journal of aircraft. 2005;42(2):413–20.

[10] Wan X. Simulation based optimization with surrogate models. Simulation based optimization with surrogate models. Ph.D. Thesis, Purdue University, 2004. Available: http://docs.lib.purdue.edu/dissertations/AAI3166722/.

[11] Rikards R, Abramovich H, Auzins J, Korjakins A. Surrogate models for optimum design of stiffened composite shells. Composite Structures. 2004; 63.2: 243–251.

[12] Hinojosa-Laborde C, Rickards CA, Ryan KL, Convertino VA. Heart Rate Variability during Simulated Hemorrhage with Lower Body Negative Pressure in High and Low Tolerant Subjects. Front Physiol. 2011;2:85 10.3389/fphys.2011.00085 22125539 PMC3221414

[13] Skillman JJ, Olson JE, Lyons JH, Moore FD. The hemodynamic effect of acute blood loss in normal man, with observations on the effect of the Valsalva maneuver and breath holding. Ann Surg. 1967;166(5):713–38. 6057082 10.1097/00000658-196711000-00001 PMC1477490

[14] Bhatt DL, Bakris GL. Renal denervation for resistant hypertension. N Engl J Med. 2014;371(2):184. 10.1056/NEJMc1405677 25006731

[15] Kandzari DE, Bhatt DL, Brar S, Devireddy CM, Esler M, Fahy M, et al Predictors of blood pressure response in the SYMPLICITY HTN-3 trial. Eur Heart J. 2015;36(4):219–27. 10.1093/eurheartj/ehu441 25400162 PMC4301597

[16] Symplicity HTNI Esler MD, Krum H Sobotka PA, Schlaich MP Schmieder RE, et al Renal sympathetic denervation in patients with treatment-resistant hypertension (The Symplicity HTN-2 Trial): a randomised controlled trial. Lancet. 2010;376(9756):1903–9. 10.1016/S0140-6736(10)62039-9 21093036

[17] Schölkopf B, Burges CJC, Smola AJ. Advances in kernel methods: support vector learning Cambridge, Mass.: MIT Press; 1999. vii, 376 p. p.

[18] Roden DM, George AL Jr. The genetic basis of variability in drug responses. Nat Rev Drug Discov. 2002;1(1):37–44. 12119608 10.1038/nrd705

[19] Wijeysundera DN, Butler GC, Ando S, Pollard M, Picton P, Floras JS. Attenuated cardiac baroreflex in men with presyncope evoked by lower body negative pressure. Clin Sci (Lond). 2001;100(3):303–9. 11222117

